# Impact of the 2024 South Brazilian flood on obstetric and mental health outcomes in pregnant women

**DOI:** 10.1007/s00737-026-01738-7

**Published:** 2026-06-20

**Authors:** Laura Paggiarin Skonieski, Pedro Giuberti, Miguel Gomes Garcia, Bernardo Bevilaqua Baldi, Sofia Turra Berlaver, Martina Alana Lodi, Gabriela Closs Machado, Egon Motyczka, Rodrigo Grassi-Oliveira, Lucas Schreiner, Thiago Wendt Viola

**Affiliations:** 1 School of Medicine, Pontifical Catholic University of Rio Grande do Sul, Porto Alegre, Brazil; 2 Grupo Hospitalar Conceição, Porto Alegre, Brazil; 3 Translational Neuropsychiatry Unit, Aarhus University, Aarhus, Denmark

**Keywords:** Climate-related disasters, Flood exposure, Perinatal mental health, Obstetric outcomes

## Abstract

**Objective:**

To examine associations between the 2024 South Brazilian flood and postpartum obstetric and maternal mental health outcomes.

**Methods:**

This cross-sectional study recruited 274 postpartum women 8–12 months after the flood at two tertiary hospitals in Porto Alegre, Brazil. We focused on three disaster-related exposures: (1) need for financial assistance due to the flood; (2) housing displacement or damage due to the flood; and (3) delivery within nine months after the flood (already pregnant at the time of the disaster).

**Results:**

Financial and housing exposures were associated with higher odds of perinatal posttraumatic stress disorder symptoms (OR = 6.7; 95% CI: 2.4–18.4 and OR = 3.6; 95% CI: 1.2–10.2, respectively) and receipt of mental health care due to the flood (OR = 6.5; 95% CI: 2.3–18.6 and OR = 5.8; 95% CI: 2.1–15.9), whereas only financial exposure was associated with perinatal depression symptoms (OR = 2.6; 95% CI: 1.03–6.6). Delivery within nine months after the disaster was associated with low birth weight (OR = 2.7; 95% CI: 1.03–7.4), gestational preeclampsia (OR = 3.4; 95% CI: 1.32–8.88), and missed prenatal appointments due to the flood (OR = 9.9; 95% CI: 2.4–39.8). Women exposed to financial or housing losses reported greater material and infrastructural disruptions, while those who delivered within nine months of the flood more frequently reported involvement in rescue/aid activities and related emotional distress.

**Conclusion:**

Disaster-related financial and housing stressors were associated with adverse maternal mental health outcomes, and delivery within nine months after the flood was associated with higher odds of adverse obstetric outcomes.

## Introduction

The United Nations Office for Disaster Risk Reduction defines a disaster as a serious disruption in the functioning of a community or society, at any scale, resulting from the interaction between hazardous events and conditions of exposure, vulnerability, and capacity, leading to human, material, economic, and environmental losses and impacts (UNDRR, 2022). In recent years, evidence indicates that disaster risk has been increasing globally, with higher numbers of deaths and affected individuals in the last five years compared to the previous period (UNDRR, 2022). This increase is directly related to climate change, as the global average temperature in 2019 was already 1.1 °C above pre-industrial levels, contributing to the intensification of the frequency and severity of extreme events, including heatwaves, droughts, floods, hurricanes, and wildfires (World Meteorological Organization 2024).

Natural disasters pose significant challenges to the provision and accessibility of healthcare services, especially in developing countries such as Brazil. Events like floods, earthquakes, and hurricanes can damage infrastructure, including healthcare facilities, making it difficult to provide care for those in need. The aftermath of these disasters can include power and communication outages, water shortages, and road damage, severely disrupting healthcare delivery (Salam et al. 2023).

The climate disaster in May 2024 in the south of Brazil, caused by torrential rains and floods, surpassed historical records. Surveys indicate that this was one of the largest natural disasters in the country’s history, with unprecedented precipitation in terms of duration, intensity, and territorial coverage (Machado 2024). The flooding generated an extreme volume of solid waste in urban areas, compromising recovery efforts for months. Several regions experienced interruptions in the supply of potable water, and multiple hospitals had to be evacuated, halting healthcare services for weeks. According to reports on the impact of the rains, of the 497 municipalities in the state of Rio Grande do Sul, 478 were affected, impacting nearly 2.4 million people. More than 15,000 km² were submerged, resulting in alarming human and social losses (ANASB, 2025; Rizzotto et al. 2024).

Pregnant people, infants, and young children face the greatest risk of adverse impacts from climate change due to biological, social, economic, and environmental factors (Weck et al. 2008). High levels of stress can arise from loss of housing, lack of water and electricity supply, and the need to relocate with family, friends, or shelters. Mobility difficulties and challenges in accessing essential healthcare, together with disruptions in prenatal care, increase health risks for these populations and their babies. Physiological and anatomical changes during pregnancy further heighten vulnerability, particularly due to modifications in thermoregulation, metabolism, as well as the cardiovascular, respiratory, and immune systems (Yüzen et al. 2023). Epidemiological studies show an association between exposure to climate disasters and adverse outcomes in pregnant women, including preterm birth, low birth weight, gestational diabetes, congenital anomalies, stillbirth, preeclampsia and psychological distress (Masters et al. 2025).

Thus, this study examines the associations between the 2024 South Brazilian flood and obstetric outcomes as well as women’s mental health indicators. Specifically, it explores three disaster-related exposures independently: (1) the need for financial assistance due to the flood, capturing economic sequelae related to the climate event; (2) housing displacement or damage attributable to the flood, reflecting direct habitat disruption and associated stressors (e.g., changes in living arrangements, reduced privacy, and prolonged uncertainty regarding return); and (3) delivery within nine months after the flood, indexing gestational timing in relation to the disaster and allowing assessment of whether pregnancies temporally proximate to the event were associated with distinct perinatal outcomes. By examining these exposure dimensions separately, the study seeks to inform the development of targeted support strategies for pregnant women in the context of future climate-related disasters.

## Materials and methods

### Design, setting, and participants

This observational cross-sectional study is nested within an ongoing prospective cohort of pregnant women and their newborns in southern Brazil established after the May 2024 floods. Clinical trial number: not applicable. Participants were recruited from December 21, 2024, to May 20, 2025, approximately 8 to 12 months after the event (last major rainfall: May 24, 2024). This window was chosen to capture women whose prenatal care was disrupted during or shortly after the floods, and women not acutely exposed before delivery but potentially experiencing persistent, disaster-related stressors and mental health sequelae.

Participants were recruited at two tertiary hospitals in Porto Alegre, the largest city in the region affected by the disaster: Hospital Nossa Senhora da Conceição and Hospital Divina Providência. Both institutions are equipped with neonatal intensive care units and routinely manage high-complexity maternal and fetal conditions, receiving referrals from across the metropolitan area. The hospitals serve women with distinct care profiles, including publicly funded care through Brazil’s Unified Health System (SUS) and private/insurance-based care, increasing socioeconomic heterogeneity in the recruited sample. Hospital Nossa Senhora da Conceição provides care exclusively through SUS, whereas Hospital Divina Providência is a private institution. Based on administrative estimates from the participating hospitals, their combined obstetric volume is approximately 450 births per month (≈ 2,250 births over a five-month period). Given that we recruited 274 women over approximately five months, the study sample represents ~ 10–12% of deliveries occurring at these institutions during the recruitment period.

On average, recruitment was feasible during approximately seven hospital shifts per week, predominantly evening shifts. Eligible women were approached at admission to the maternity unit, and the enrollment acceptance rate exceeded 90%. A convenience sampling strategy was employed, inviting all eligible women admitted for delivery during the periods when research team members were present. The study protocol received ethics approval from all participating institutions and was conducted in accordance with national and institutional regulations governing research involving human participants.

### Eligibility criteria

Inclusion criteria were: women aged 18 to 45 years, admitted for childbirth at the participating hospitals during the recruitment period, and able to provide informed consent. There was no exclusion criteria related to clinical conditions. The only exclusion applied when a participant could not complete the interview/questionnaires due to difficulty or confusion at the time of assessment (precluding valid data collection and consent).

### Sociodemographic and clinical data

Maternal characteristics included age, income, education, parity, body mass index (BMI), and ethnicity. Obstetric and neonatal information were abstracted from medical records by trained nurses or medical residents using a standardized data extraction form, and included admission to intensive care, preterm birth, low birth weight, gestational preeclampsia (PE), gestational diabetes mellitus (DM), high-risk pregnancy, and missed prenatal appointments attributable to the flood. Perinatal mental health indicators and flood-related consequences were assessed by trained psychologists within 48 h postpartum during the hospital stay, using standardized questionnaires and interview protocols. When women were discharged before the in-hospital assessment could be completed, they were contacted by telephone within three weeks postpartum to complete the same instruments, ensuring consistency of data collection across modes.

### Mental-health assessment

Perinatal major depressive episode was assessed with the Beck Depression Inventory (BDI-II), a 21-item self-report instrument that evaluates depressive symptom severity over the past two weeks; each item is scored from 0 to 3, yielding a total score range of 0–63 (Inventário de depressão de beck - II, 2021). We used the severe cutoff 29 points to classify cases (Wang and Gorenstein 2013). Posttraumatic stress disorder (PTSD) symptoms were measured with the Posttraumatic Stress Disorder Checklist for DSM-5 (PCL-5) (Lima et al., 2016), a 20-item self-report measure of DSM-5 PTSD symptoms rated from 0 (“not at all”) to 4 (“extremely”), with total scores ranging from 0 to 80. Scores > 33 were used to indicate probable PTSD (Bovin et al. 2016). Mental-health assistance during pregnancy due to the flood consequences was ascertained by self-report.

### Flood-exposure assessment

Flood impacts were measured with a structured interview developed by the authors to capture key consequences of the disaster: duration of residential displacement, severity of water-related housing damage, days without water supply, days without electricity, perceived financial impact, time spent in rescue/aid activities, and emotional distress during those activities. Items used ordinal Likert-type scales:

- Duration of residential displacement (0–5): 0 = did not leave residence; 1 = < 1 week; 2 = 1–2 weeks; 3 = 2–4 weeks; 4 = 1–2 months; 5 = > 2 months.
- Severity of water-related housing damage (0–3): 0 = no damage; 1 = minor damage (repairable with small adjustments); 2 = moderate damage (requiring significant repairs); 3 = severe damage (requiring full reconstruction).
- Duration without water supply (0–4): 0 = no interruption; 1 = < 24 h; 2 = < 1 week; 3 = > 1 week; 4 = > 1 month.
- Duration without power supply (0–4): 0 = no interruption; 1 = < 24 h; 2 = < 1 week; 3 = > 1 week; 4 = > 1 month.
- Perceived financial impact (0–4): 0 = no financial impact; 1 = mild reduction in household income; 2 = moderate reduction; 3 = severe reduction; 4 = complete loss of household income.
- Time spent in rescue/aid activities (0–5): 0 = did not engage in any activity; 1 = < 1 week; 2 = 1–2 weeks; 3 = 2–4 weeks; 4 = 1–2 months; 5 = > 2 months.
- Emotional distress during rescue/aid activities (0–4): 0 = did not engage in any activity; 1 = no distress; 2 = low; 3 = moderate; 4 = high.
- Pregnancy-related exposure was operationalized as an ordinal variable based on time from the disaster to birth: ≤9 months, 10–12 months, and > 12 months after the event.

The ordinal items were used to derive the binary exposures and kept in ordinal form for the dose-response and severity analyses: (1) Financial consequences, where participants were classified as financially exposed if they needed/received financial assistance owing to the flood (from government, community organizations, or family/friends). All forms of economic support reported, irrespective of income level, were included in this classification. Examples include *Auxílio Reconstrução*, a federal program that provided a one-time payment of R$5,100.00 to families affected by the floods who were displaced, either temporarily or permanently, from areas officially declared under a state of calamity, and *Programa Volta por Cima*, a state initiative granting R$2,000.00 to households impacted by extreme weather events, such as the May 2024 floods. Both programs were implemented in May 2024; (2) Housing displacement/damage, where participants were classified as housing-exposed if they either left and stayed away from their residence for ≥ 1 month (categories 4–5 on the displacement scale) or reported water-related housing damage = 3 (severe); and (3) A pregnancy exposure domain for births occurring within nine months after the event, referred to women who were already pregnant at the time of the disaster (up to February 15, 2025).

### Statistical analysis

All dependent variables were modeled as dichotomous outcomes (0 = no/absence; 1 = yes/presence). The obstetric outcomes were: neonatal intensive care unit (NICU) admission (yes/no), preterm birth (< 37 weeks of gestation; yes/no), low birth weight (< 2500 g; yes/no), gestational PE (yes/no), gestational DM (yes/no), and missed prenatal care appointment(s) due to the flood (yes/no). Gestational PE and gestational DM corresponded to clinical diagnoses identified during the index pregnancy. The maternal mental health outcomes were: perinatal major depressive episode symptoms (yes/no), perinatal PTSD symptoms (yes/no), and receipt of mental health care during pregnancy due to the flood (yes/no). Perinatal depressive symptoms and PTSD symptoms were assessed using the BDI and the PCL-5, respectively, and dichotomized according to the study’s predefined thresholds. Receipt of mental health care during pregnancy due to the flood was ascertained via self-report during the study interview.

We used binary logistic regression models to estimate odds ratios (ORs) and 95% confidence intervals (95% CIs) for the associations between each exposure domain (housing displacement/damage, need for financial assistance, and delivery within 9 months after the event) and each binary perinatal outcome (maternal mental health indicators and obstetric outcomes). We also fitted multivariable binary logistic regression models adjusting for maternal age, BMI, number of previous gestations, White race/skin color, education level, and insurance type (private or SUS) as a proxy for socioeconomic level. Household income was not included as a covariate because 15 participants had missing data, which would have reduced the analytic sample. Adjusted models were estimated separately for each outcome, and we also report the Hosmer-Lemeshow goodness-of-fit test (p-values), with non-significant results suggesting acceptable model fit. To account for the increased risk of Type I error arising from multiple logistic regression models across six obstetric and three mental health outcomes, adjusted p-values were computed using the Benjamini-Hochberg false discovery rate (FDR) procedure, applied independently for each exposure group (financial, housing, and conception) and for each outcome domain (obstetric and mental health).

Supplementary dose-response analyses were conducted using ANOVA to compare mean values of continuous variables across ordered exposure levels and, for associations involving ordinal exposure categories and categorical outcomes, the linear-by-linear association test to assess monotonic trends across increasing exposure levels.

To compare the average severity of flood consequences across exposure groups (housing, financial, pregnancy), independent-samples t-tests were conducted for six continuous variables: duration of displacement, days without water, days without electricity, perceived financial impact, time spent in rescue/aid activities, and emotional distress during rescue/aid activities. The analyses were performed using IBM SPSS Statistics v.20 with a statistical significance threshold of *p* < 0.05.

## Results

### Sample description

A total of 274 pregnant women were included in the study (Table 1). The mean maternal age was 27.7 years (SD = 6.0), 64% were White participants (*n* = 177), the mean number of previous pregnancies was 1.2, and the mean BMI was 27.5 (SD = 7.1). Regarding obstetric and neonatal outcomes, 17.5% of newborns required NICU admission, 16.0% were preterm, and 8.0% had low birth weight. The prevalence of gestational preeclampsia and gestational diabetes was 8.5% and 16.0%, respectively, while 51.5% of pregnancies were classified as high-risk. In relation to mental health, 8.6% of participants scored on the BDI consistent with a probable major depressive episode, 8.0% scored on the PCL indicating probable PTSD, and 7.5% reported receiving mental health assistance during pregnancy due to the flood.

**Table 1:** Sample’s description (n = 274)

Variables	(*n*) / mean	(%) / SD
Maternal characteristics		
Age	27.7	6.0
Income^a^	2.6	1.1
Education^b^	4.0	1.7
Previous gestations	1.2	1.4
BMI	27.5	7.1
White race/skin color	177	(64.4)
Insurance type - Private	34	(12.5)
Obstetric outcomes		
NICU admission	(47)	(17.5)
Preterm	(43)	(16.0)
Low birth weight (< 2500 g)	(21)	(8.0)
Gestational PE	(23)	(8.5)
Gestational DM	(43)	(16.0)
High-risk pregnancy	(139)	(51.5)
Missed prenatal care appointment due to the flood	(16)	(6)
Perinatal major depression episode	(23)	(8.6)
Perinatal PTSD	(21)	(8.0)
Mental health assistance during pregnancy due to the flood	(20)	(7.5)
Flood-related consequences		
Residential displacement^c^	0.8	1.6
Water supply interruption^d^	2.4	1.2
Power supply interruption^e^	1.6	1.5
Financial impact^f^	0.8	1.1
Rescue and aid activities involvement^g^	1.3	1.6
Emotional distress experienced during rescue/aid activities^h^	1.7	1.8

PE – Pre-eclampsia. DM – Diabetes Mellitus. Flood-related consequence variables (footnotes C–H) are reported as means (SD) of ordinal scale scores; scale labels are described in the corresponding footnotes. A – Income (monthly household income, BRL): 1 = < R 1,412–2,999; 3 = R 5,000–9,999; 5 = ≥ R$ 10,000. B – Education: 1 = Incomplete primary school; 2 = Completed primary school; 3 = Incomplete high school; 4 = Completed high school; 5 = Technical/Vocational training; 6 = Incomplete higher education; 7 = Completed higher education. C – Residential displacement: 0 = did not leave residence; 1 = < 1 week; 2 = 1–2 weeks; 3 = 2–4 weeks; 4 = 1–2 months; 5 = > 2 months. D – Water supply interruption: 0 = no interruption; 1 = < 24 h; 2 = < 1 week; 3 = > 1 week; 4 = > 1 month. E – Power supply interruption: 0 = no interruption; 1 = < 24 h; 2 = < 1 week; 3 = > 1 week; 4 = > 1 month. F – Financial impact: 0 = no financial impact; 1 = mild reduction in household income; 2 = moderate reduction; 3 = severe reduction; 4 = complete loss of household income. G – Rescue/aid activities involvement: 0 = did not engage in any activity; 1 = < 1 week; 2 = 1–2 weeks; 3 = 2–4 weeks; 4 = 1–2 months; 5 = > 2 months. H – Emotional distress during rescue/aid activities: 0 = did not engage in any activity; 1 = no emotional distress; 2 = low; 3 = moderate; 4 = high

With respect to disaster-related exposure domains, 20.1% (*n* = 55) had housing displacement/damage, 21.9% (*n* = 60) had financial-need exposure, and 28.5% (*n* = 78) had pregnancy-timing exposure (delivery within nine months of the event). Only 11 cases were exposed to the three types of exposures simultaneously.

### Flood-related consequences on obstetrical and mental health outcomes

In the adjusted logistic regression models, financial exposure (Table 2) was strongly associated with perinatal mental health outcomes, with significant associations observed for perinatal major depression symptoms (OR = 2.6, 95% CI: 1.03–6.69), perinatal PTSD symptoms (OR = 6.7, 95% CI: 2.45–18.4), and receipt of mental health care during pregnancy (OR = 6.5, 95% CI: 2.31–18.6). Housing exposure (Table 3) was linked to higher odds of perinatal PTSD symptoms (OR = 3.6, 95% CI: 1.28–10.2), and mental-health assistance during pregnancy (OR = 5.8, 95% CI: 2.15–15.9). By contrast, neither financial nor housing exposure showed significant associations with obstetric outcomes, including NICU admission, preterm birth, low birth weight, gestational PE or DM.

**Table 2:** Associations between financial exposure with obstetric outcomes

Exposure characterized as financial need assistance due to the disaster	Exposed (*n* = 60)	Not-exposed (*n* = 214)	Unadjusted		Adjusted			
Obstetric outcomes	n (%)	n (%)	OR (CI)	p-val	OR (CI)	p-val	FDR	HL
NICU admission	12 (20.3)	35 (16.7)	1.2 (0.61–2.65)	0.51	1.2 (0.59–2.74)	0.52	0.62	0.54
Preterm	9 (15.3)	34 (16.3)	0.9 (0.41–2.06)	0.85	0.8 (0.43–1.67)	0.43	0.62	0.90
Low birth weight (< 2500 g)	2 (3.5)	19 (9.2)	0.3 (0.08–1.59)	0.17	0.3 (0.07–1.60)	0.17	0.62	0.40
Gestational PE	8 (13.3)	15 (7.0)	2.0 (0.82–5.07)	0.12	1.6 (0.59–4.38)	0.35	0.62	0.52
Gestational DM	13 (21.7)	30 (14.0)	1.7 (0.82–3.50)	0.15	1.5 (0.65–3.42)	0.33	0.62	0.95
Missed prenatal care appointment due to the flood	3 (5.0)	13 (6.1)	0.8 (0.22–2.95)	0.75	0.8 (0.20–3.26)	0.77	0.77	0.06
Mental health outcomes								
Perinatal major depression episode	10 (16.9)	13 (6.2)	3.0 (1.27–7.43)	0.01*	2.6 (1.03–6.69)	0.04*	0.04*	0.55
Perinatal PTSD	13 (22.0)	8 (3.8)	7.1 (2.79–18.21)	0.00*	6.7 (2.45–18.44)	0.00*	0.00*	0.75
Mental health assistance during pregnancy due to the flood	10 (16.7)	10 (4.7)	4.0 (1.61–10.33)	0.00*	6.5 (2.31–18.61)	0.00*	0.00*	0.92

NICU - Neonatal Intensive Care Unit. PE - Pre-eclampsia. DM - Diabetes Mellitus. CI – 95% confidence intervals. Each row represents an independent logistic regression model testing the association between the specified exposure and outcome, adjusted for maternal age, maternal BMI, previous gestations, maternal educational level, White race/skin color and insurance type. HL - Hosmer–Lemeshow test p-value. * Indicates significant associations. FDR – Benjamini-Hochberg false discovery rate adjusted p-value, computed independently within each outcome domain (obstetric and mental health)

**Table 3:** Associations between housing exposure with obstetric outcomes

Exposure characterized as housing displacement/damage due to the disaster	Exposed (*n* = 55)	Not-exposed (*n* = 219)	Unadjusted		Adjusted			
Obstetric outcomes	n (%)	n (%)	OR (CI)	p-val	OR (CI)	p-val	FDR	HL
NICU admission	13 (24.1)	34 (15.8)	1.6 (0.81–3.48)	0.15	1.8 (0.86–3.80)	0.11	0.66	0.09
Preterm	9 (17.0)	34 (15.8)	1.0 (0.48–2.43)	0.83	0.7 (0.36–1.51)	0.41	0.86	0.43
Low birth weight (< 2500 g)	2 (3.9)	19 (8.9)	0.4 (0.09–1.85)	0.25	0.4 (0.09–1.98)	0.43	0.86	0.42
Gestational PE	5 (9.1)	18 (8.2)	1.1 (0.39–3.15)	0.83	0.89 (0.30–2.61)	0.83	0.89	0.58
Gestational DM	10 (18.2)	33 (15.1)	1.2 (0.57–2.72)	0.57	0.9 (0.37–2.15)	0.81	0.89	0.38
Missed prenatal care appointment due to the flood	3 (5.5)	13 (5.9)	0.9 (0.25–3.32)	0.89	0.9 (0.24–3.45)	0.89	0.89	0.24
Mental health outcomes								
Perinatal major depression episode	9 (16.7)	14 (6.5)	1.8 (0.72–4.74)	0.20	1.6 (0.59–4.66)	0.33	0.33	0.06
Perinatal PTSD	11 (20.4)	10 (4.6)	5.2 (2.09–13.12)	0.00*	3.6 (1.28–10.21)	0.01*	0.02*	0.18
Mental health assistance during pregnancy due to the flood	10 (18.2)	10 (4.6)	4.6 (1.82–11.81)	0.00*	5.8 (2.15–15.99)	0.00*	0.00*	0.27

NICU - Neonatal Intensive Care Unit. PE - Pre-eclampsia. DM - Diabetes Mellitus. CI – 95% confidence intervals. Each row represents an independent logistic regression model testing the association between the specified exposure and outcome, adjusted for maternal age, maternal BMI, previous gestations, maternal educational level, White race/skin color and insurance type. HL - Hosmer–Lemeshow test p-value. * Indicates significant associations. FDR – Benjamini-Hochberg false discovery rate adjusted p-value, computed independently within each outcome domain (obstetric and mental health)

Delivery within nine months of the event (Table 4) was associated with higher odds of low birth weight (OR = 2.7, 95% CI: 1.03–7.48) and gestational PE (OR = 3.4, 95% CI: 1.32–8.88). It was also strongly associated with missed prenatal appointments due to the flood (OR = 9.9, 95% CI: 2.47–39.87). No significant association was observed for NICU admission, preterm birth, gestational DM, nor for perinatal major depression, perinatal PTSD, or receipt of mental-health assistance during pregnancy.

**Table 4:** Associations between conception exposure with obstetric outcomes

Exposure characterized as births occurring within 9 months of the event	Exposed (*n* = 78)	Not-exposed (*n* = 196)	Unadjusted		Adjusted			
Obstetric outcomes	n (%)	n (%)	OR (CI)	p-val	OR (CI)	p-val	FDR	HL
NICU admission	15 (20.5)	32 (16.3)	1.4 (0.76–2.87)	0.24	1.3 (0.66–2.72)	0.41	0.49	0.56
Preterm	14 (19.4)	29 (14.8)	1.3 (0.67–2.69)	0.39	1.4 (0.79–2.63)	0.23	0.35	0.63
Low birth weight (< 2500 g)	10 (13.7)	11 (5.8)	2.4 (1.02–5.96)	0.04*	2.7 (1.03–7.48)	0.04*	0.08	0.60
Gestational PE	11 (14.1)	12 (6.1)	2.5 (1.07–6.00)	0.03*	3.4 (1.32–8.88)	0.01*	0.03*	0.59
Gestational DM	9 (11.5)	34 (17.3)	0.6 (0.28–1.34)	0.22	0.7 (0.45–2.81)	0.78	0.78	0.15
Missed prenatal care appointment due to the flood	12 (15.4)	4 (2.0)	8.7 (2.72–27.99)	0.00*	9.9 (2.47–39.87)	0.00*	0.00*	0.44
Mental health outcomes								
Perinatal major depression episode	6 (8.2)	17 (8.7)	0.9 (0.35–2.47)	0.89	1.5 (0.52–4.29)	0.44	0.99	0.95
Perinatal PTSD	5 (6.8)	16 (8.2)	0.8 (0.28–2.29)	0.69	0.9 (0.30–3.30)	0.99	0.99	0.13
Mental health assistance during pregnancy due to the flood	7 (9.0)	13 (6.6)	1.3 (0.53–3.62)	0.50	1.1 (0.40–2.92)	0.86	0.99	0.20

NICU - Neonatal Intensive Care Unit. PE - Pre-eclampsia. DM - Diabetes Mellitus. CI – 95% confidence intervals. Each row represents an independent logistic regression model testing the association between the specified exposure and outcome, adjusted for maternal age, maternal BMI, previous gestations, maternal educational level, White race/skin color and insurance type. HL - Hosmer–Lemeshow test p-value. * Indicates significant associations. FDR – Benjamini-Hochberg false discovery rate adjusted p-value, computed independently within each outcome domain (obstetric and mental health)

When FDR-adjusted p-values were applied, all significant associations remained, with the exception of low birth weight among deliveries within nine months of the event, for which the FDR-adjusted p-value increased to 0.08.

### Dose-response relationships

**Fig. 1:**
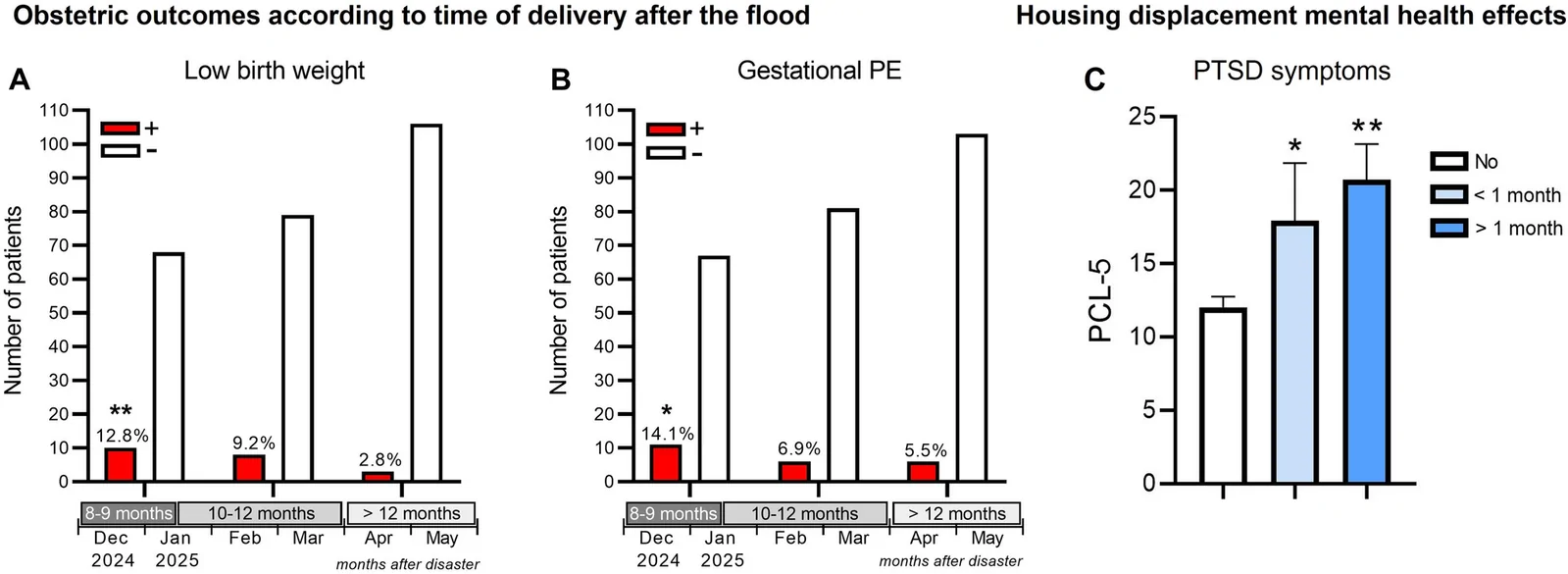
(**A**–**B**) Obstetric outcomes according to time of delivery after the flood (proxy for temporal proximity to the disaster), categorized as ≤ 9 months, 10–12 months, and > 12 months after the event. Bars show the number of women with the outcome present (+, colored bars) and absent (−, open bars); percentages indicate the proportion of women with the outcome in each category. Trend associations across ordered time-since-disaster categories were tested using the linear-by-linear association test. (**C**) Mean PCL-5 scores according to duration of housing displacement (no displacement, < 1 month, > 1 month). Bars represent mean values and error bars indicate standard error. Asterisks denote statistical significance (* *p* < 0.05; ** *p* < 0.01)

We conducted supplementary dose-response analyses using non-binarized exposure measures to examine whether obstetric risks varied with temporal proximity to the flood. Pregnancy-related exposure was operationalized as an ordinal variable based on time from the disaster to birth: ≤9 months (*n* = 78), 10–12 months (*n* = 87), and > 12 months after the event (*n* = 109). These ordered categories were examined in relation to the binary obstetric outcomes of low birth weight and gestational PE. The linear-by-linear association test indicated significant trend associations (Fig. 1A-B), with higher frequencies of low birth weight (12.8%, 9.2%, 2.8%) and gestational PE (14.1%, 6.9%, 5.5%) among births occurring closer to the disaster (low birth weight: χ² = 6.74, *p* = 0.009; PE: χ² = 4.08, *p* = 0.043).

To further examine dose-response patterns regarding mental health outcomes, we compared PCL-5 scores across ordered housing displacement groups, an analysis focused on PTSD symptoms. Participants were classified as no housing displacement (*n* = 206), mild exposure (displacement < 1 month; *n* = 21), or moderate-to-severe exposure (displacement > 1 month; *n* = 43). One-way ANOVA indicated significant differences in PCL-5 scores between groups (F(2,267) = 10.17, *p* < 0.001; Fig. 1C). Post-hoc comparisons (LSD) showed that both the mild exposure group and the moderate-to-severe exposure group had significantly higher PCL-5 scores than the non-displaced group (mean difference = 5.90, 95% CI: 0.35–11.44, *p* = 0.037; and mean difference = 8.71, 95% CI: 4.65–12.77, *p* < 0.001, respectively). In contrast, PCL-5 scores did not differ significantly between the mild and moderate-to-severe exposure groups (mean difference = 2.82, 95% CI: −3.63 to 9.26, *p* = 0.390). We also compared BDI-II scores across perceived flood-related financial impact categories using ANOVA, but no statistically significant differences were observed.

### Severity of flood-related consequences

**Fig. 2:**
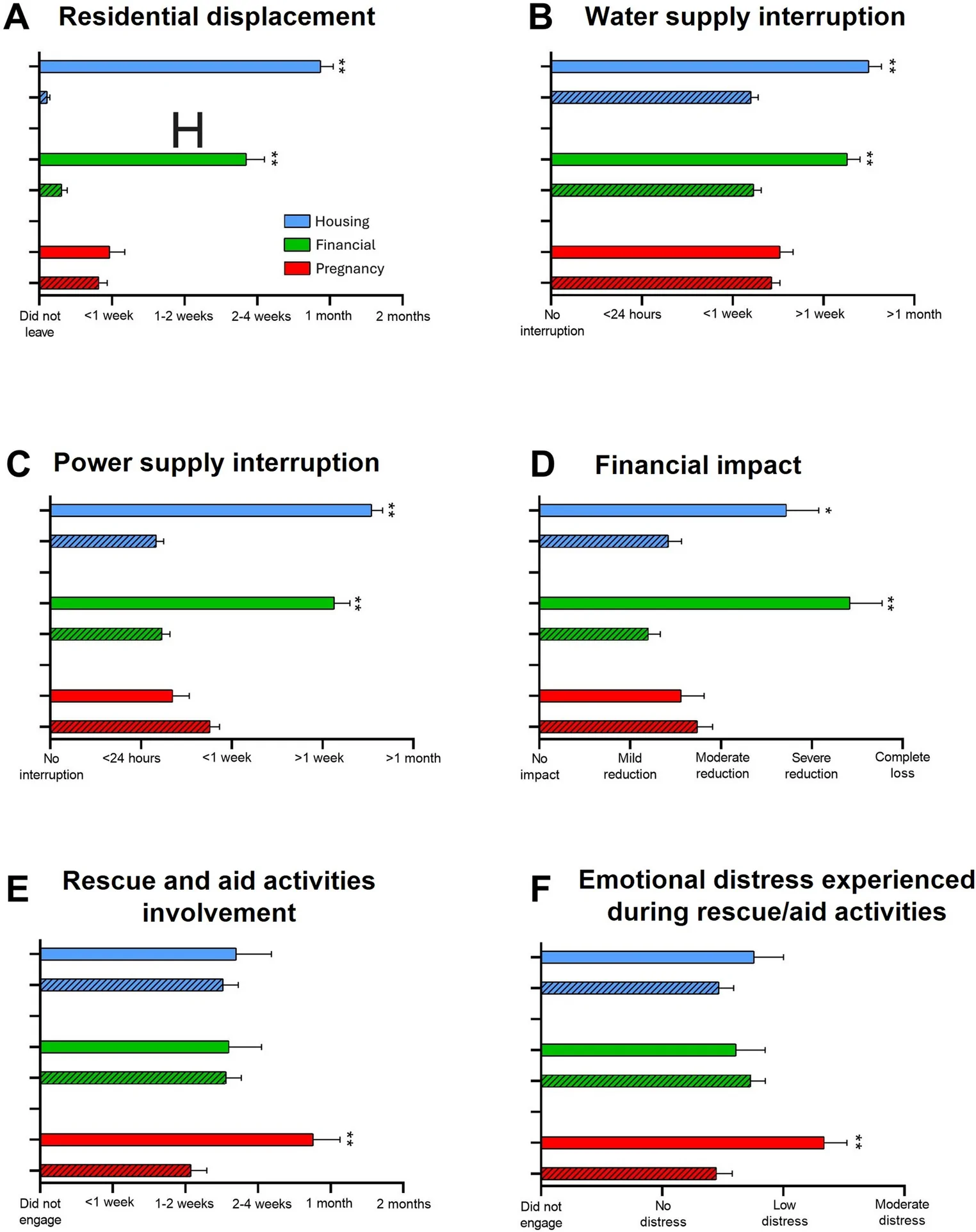
Solid bars represent the group exposed to the specific flood-related situation, while dashed bars represent the corresponding reference (non-exposed) group. (**A**) duration of residential displacement, (**B**) duration without water supply, (**C**) duration without power supply, (**D**) perceived financial impact, (**E**) time spent in rescue and aid activities, and (**F**) emotional distress during rescue/aid activities. Error bars represent standard errors. * = *p* < 0.05. ** = *p* < 0.01

Figure 2 illustrates how the three exposure domains (housing, financial, and pregnancy) were related to six dimensions of flood-related consequences. Women in the housing exposure group reported significantly higher scores for residential displacement (Fig. 2A; mean 4.07 vs. 0.06), water supply interruption (Fig. 2B; mean 3.55 vs. 2.19), and power supply interruption (Fig. 2C; mean 3.53 vs. 1.18) compared to non-exposed women (*p* < 0.01 for all). They also reported higher financial impact scores (Fig. 2D; mean 1.51 vs. 0.68). Similarly, the financial exposure group reported higher scores than non-exposed women for residential displacement (mean 2.85 vs. 0.31), water supply interruption (mean 3.27 vs. 2.24), power supply interruption (mean 3.13 vs. 1.23), and financial impact (mean 1.72 vs. 0.60; Fig. 2A–D; all p-values < 0.01). In contrast, the delivery-within-nine-months group showed higher scores for rescue/aid activities involvement (mean 1.88 vs. 1.05) and emotional distress during rescue/aid activities (mean 2.35 vs. 1.45) compared to the non-exposed group (Fig. 2E–F).

## Discussion

This study examines the associations between the 2024 South Brazilian flood for both obstetric and perinatal mental health outcomes. We found that financial and housing exposures were consistently associated with poorer perinatal mental health, including higher odds of major depression symptoms, PTSD symptoms, and receipt of mental-health assistance during pregnancy. In contrast, delivery within nine months after the disaster was not related to mental health outcomes, but was associated with adverse obstetric indicators, including low birth weight, gestational pre-eclampsia, and missed prenatal appointments. These associations remained significant after adjustment for maternal age, BMI, educational level, White race/skin color and insurance type. Furthermore, analyses of flood-related consequences highlighted that women exposed to housing and financial losses reported greater material and infrastructural disruptions, whereas women who delivered within nine months of the flood were more directly involved in rescue and aid activities and reported higher levels of emotional distress while performing these activities.

The finding that women who were pregnant at the time of the flood event experienced statistically significantly higher rates of gestational PE is consistent with previous research indicating that flooding may increase the risk of hypertensive disorders by altering environmental exposures. In New Orleans, for example, pregnant women living in neighborhoods with high soil lead levels had up to fourfold higher odds of eclampsia, whereas areas where flooding reduced soil lead concentrations subsequently showed a decline in cases (Zahran et al. 2014). Furthermore, biological stress responses, such as elevated cortisol levels, have been associated with adverse pregnancy outcomes such as PE, which may partially explain our results (Caparros-Gonzalez et al. 2022).

Another important finding was that women who were pregnant at the time of the floods had significantly higher rates of low birth weight (< 2500 g). Previous studies indicate that environmental disasters disproportionately affect maternal health by limiting access to healthcare services and exacerbating nutritional insecurity (Kota et al. 2025). Reduced access to prenatal care during disasters, which in our study was also associated with pregnancy exposure, may delay the diagnosis and management of complications that compromise fetal growth, thereby increasing the likelihood of adverse birth outcomes. In addition, food scarcity and disruption of nutritional programs during and after climate disasters may impair maternal nutrition and fetal development. Beyond healthcare access and nutrition, acute biological stress responses to disaster-related consequences, such as elevated cortisol levels, have been consistently linked to intrauterine growth restriction and low birth weight (Bolten et al. 2011). Therefore, these findings suggest that the increased risk of low birth weight among women pregnant during the flood likely reflects the combined influence of social, environmental, and biological stressors. However, the higher odds of low birth weight observed in both unadjusted and adjusted models did not survive FDR correction (FDR-adjusted *p* = 0.08) and should therefore be interpreted with caution.

Furthermore, the supplementary dose-response analyses provided additional evidence that disaster-related exposures may be patterned by intensity and timing. First, the increase in the frequency of low birth weight and gestational PE among births occurring closer to the flood is consistent with a temporal-proximity gradient, whereby pregnancies whose late gestation overlapped more directly with the acute disaster phase may have experienced greater disruption to prenatal care, heightened psychosocial stress, and potential physiological stress responses relevant to hypertensive disorders of pregnancy and fetal growth. Although these trend tests do not establish causality, they suggest that the acute and immediate post-disaster period may represent a particularly vulnerable window for obstetric risk.

Notably, women who delivered within nine months after the disaster were also more frequently engaged in aid and rescue activities, which may account for their higher reports of emotional distress related to these experiences. However, these findings should be interpreted with caution, as greater distress associated with such involvement did not increase their risk of developing perinatal major depression or perinatal PTSD symptoms. Previous research with post-disaster volunteers in Ōtautahi Christchurch, Aotearoa New Zealand, similarly demonstrated that engagement in community response efforts generated significant well-being benefits, which were instrumental in helping participants counter the potentially negative psychological impacts of the disaster (Carlton and Wong 2024). Although many volunteers reported exhaustion and emotional strain, particularly those involved in the immediate aftermath of the event, they also described a sense of purpose, belonging, and fulfillment derived from contributing to collective recovery and supporting others. These findings align with the present study, suggesting that although participation in rescue and aid activities may elicit heightened emotional arousal, such engagement can also promote resilience and protect against psychological distress, thereby reducing the likelihood of developing depression and PTSD.

Beyond the higher risk of gestational PE and low birth weight, our findings are consistent with research from Hurricane Harvey in the United States, which also found no significant association between flood exposure and spontaneous preterm birth (Liu et al. 2024). One possible explanation is that preterm birth may be less directly influenced by acute disaster-related stressors compared to outcomes such as hypertensive disorders or fetal growth restriction, which are more sensitive to nutritional deficits, toxic environmental exposures, and maternal stress physiology.

Despite the obstetric findings, our results indicate that women facing financial impact, homelessness or housing damage due to the flood had higher rates of major depression symptoms, perinatal PTSD symptoms, and a greater need for mental health assistance during pregnancy. A systematic review by Harville et al. (2010) demonstrated that exposure to natural disasters, particularly when accompanied by displacement and housing instability, is consistently associated with increased maternal psychological distress, including depression and post-traumatic stress symptoms. Moreover, the unpredictability and lack of control inherent in displacement intensify feelings of helplessness and vulnerability, which are core characteristics of psychological traumatic experiences associated with PTSD (Davis et al. 2010). Indeed, PCL-5 scores were higher among women who experienced housing displacement, both for displacement < 1 month and > 1 month, compared with those not displaced, suggesting that displacement itself may be a salient stressor associated with increased PTSD symptom burden, and we also observed that mean scores were numerically higher in the > 1 month group.

Economic losses resulting from the flood also had important effects on maternal mental health. Our results indicate that women who experienced financial hardship during the floods were more likely to present perinatal major depression symptoms, perinatal PTSD symptoms and the need for mental health assistance during pregnancy due to the flood compared with those who were not exposed. Previous literature indicate that economic instability during pregnancy is a documented risk factor for antenatal anxiety and depression (Verbeek et al. 2019). Importantly, even in the multivariable logistic regression models, the adjusted associations between financial exposure and both depression and PTSD symptoms remained significant, suggesting that financial losses represent a key pathway through which climate disasters contribute to perinatal depressive symptoms. These findings reinforce the importance of integrating economic support, such as emergency subsidies, and mental health support into disaster response frameworks to protect maternal and fetal health.

This study has important limitations. First, recruitment was based on convenience sampling, restricted to periods when research team members were available during hospital shifts. Although this represents a limitation, it should be noted that both recruitment and data collection were performed during the puerperium, and the acceptance rate for enrollment was above 90%. Second, flood-related exposures and consequences were assessed through a questionnaire developed by the authors. While this approach is subject to recall bias due to self-report, recruitment was concluded within 12 months after the climate disaster and, given the unprecedented consequences for most of the population in Rio Grande do Sul, it is unlikely that participants provided highly imprecise responses. To further mitigate this issue, Likert-type scales were used to capture severity across interval ranges of exposure rather than requiring exact estimations. Third, only 11 participants met all three criteria of exposures (financial constraints, housing constraints, and conception/pregnancy exposure), which precluded a statistical analysis of cumulative or synergistic effects in the present sample. Fourth, although significant associations were observed between disaster-related exposures and psychological symptoms, the temporal direction of these relationships cannot be definitively established, longitudinal studies are needed to clarify temporal relationships and potential causal pathways. Fifth, the absence of a non-disaster comparison group or pre-event baseline assessed with the same protocol, precludes direct evaluation of whether the sample characteristics differ from those of pregnant women in non-disaster periods. Sixth, because we did not have individual-level information on women who declined participation, we could not compare participants and non-participants on observable characteristics; therefore, non-response bias cannot be ruled out. Seventh, the sample comprised 51% high-risk pregnancies, recruited from two tertiary referral hospitals with neonatal intensive care units, Hospital Divina Providência and Hospital Nossa Senhora da Conceição, which routinely manage complex maternal and fetal conditions. As referral centers, these institutions typically receive women with pre-existing comorbidities, obstetric complications, and pregnancies requiring specialized perinatal care, which likely contributes to the higher proportion of high-risk classifications observed in our sample compared to general obstetric populations. Despite these limitations, we successfully recruited more than 270 women in the puerperium and identified important associations between distinct types of flood-related exposures and adverse obstetric and mental health outcomes.

## Conclusion

In conformity with the Sustainable Development Goals (SDGs) of the United Nations 2030 Agenda, the Sendai Framework for Disaster Risk Reduction (2015–2030) establishes international standards for preventing and mitigating the effects of climate-related events. While the Sendai Framework outlines specific actions such as understanding risks, strengthening governance, investing in resilience, and improving response, the SDGs incorporate these objectives into a broader agenda for human, economic, and environmental development. Together, they promote safer and more sustainable societies, recognizing disaster risk reduction as essential for eradicating poverty, protecting health, ensuring education, and addressing climate change (UNDRR, 2022).

In this context, government measures aimed at protecting vulnerable populations, particularly pregnant women, are critical to reducing the impacts of climate-related disasters. Such measures should include the development of early warning systems, guaranteeing uninterrupted access to prenatal care during crises through mobile health units or telemedicine, and distributing emergency kits with prenatal medications, safe drinking water, and appropriate food for pregnant women. Moreover, immediate and long-term psychological support policies, along with shelter programs that prioritize families with pregnant women, can mitigate adverse maternal and fetal health outcomes. Finally, once a flood has occurred, policies that specifically reduce housing instability and financial losses among pregnant women are essential to prevent perinatal mental health problems, thus protecting both maternal resilience and well-being, as well as child development.

## Data Availability

Data will be available upon request.
